# Supplementary material for: Inflammation causes insulin resistance in mice via interferon regulatory factor 3 (IRF3)-mediated reduction in FAHFA levels
Source: Nat Commun. 2024 May 30;15:4605. doi: 10.1038/s41467-024-48220-5 (PMC11139994; doi:10.1038/s41467-024-48220-5)
Supplement: Supplementary file 1 — Supplementary Information [file 41467_2024_48220_MOESM1_ESM.pdf]

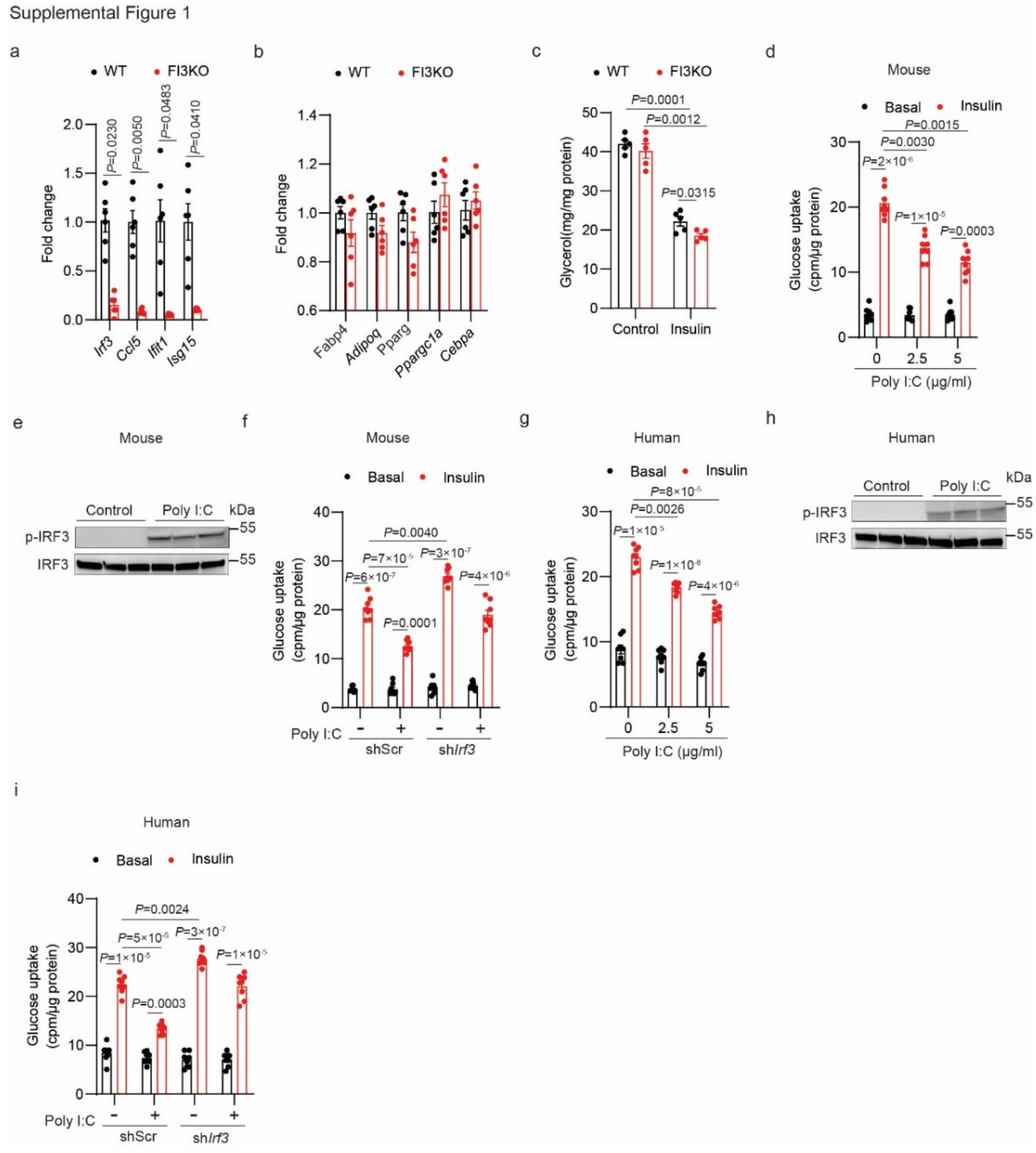

Supplementary Fig. 1. IRF3 is necessary for TLR3-mediated insulin resistance in mouse and human adipocytes. **a**, mRNA analysis of *Irf3*, *Ccl5*, *Ifit1*, and *Isg15* in WT and FI3KO SVF-derived adipocytes (n=6). **b**, mRNA analysis of *Fabp4*, *Adipoq*, *Pparg*, *Ppargc1a*, and *Cebpa* in WT and FI3KO SVF-derived adipocytes (n=5). **c**, Lipolysis in WT and FI3KO SVF-derived adipocytes treated with or without insulin (10nM, 4h) (n=5). **d**, Glucose uptake in mouse adipocytes after treatment with varying Poly I:C doses for 2 days (n=8). **e**, Western blot showing phosphorylation of murine IRF3 (S388) in mouse adipocytes after 30 mins of Poly I:C (5 μg/ml) treatment. **f**, Glucose uptake in mouse adipocytes transduced with lentivirus expressing shRNA against Irf3 or shScr control hairpin in the absence or presence of Poly I:C (5 μg/ml) (n=8). **g**, Glucose uptake in human adipocytes after treatment with varying Poly I:C doses for 2 days (n=8). **h**, Western blot showing phosphorylation of human IRF3 (S396) in human adipocytes after 30 mins of Poly I:C (5 μg/ml) treatment. **i**, Glucose uptake in human adipocytes transduced with lentivirus expressing shRNA against Irf3 or shScr control hairpin in the absence or presence of Poly I:C (5 μg/ml) (n=8). Statistical significance was assessed by *two-way* ANOVA (**c**, **d** and **g**) or *three-way* ANOVA (**f** and **i**). Data in all panels are expressed as mean ± SEM.

Supplemental Figure 2

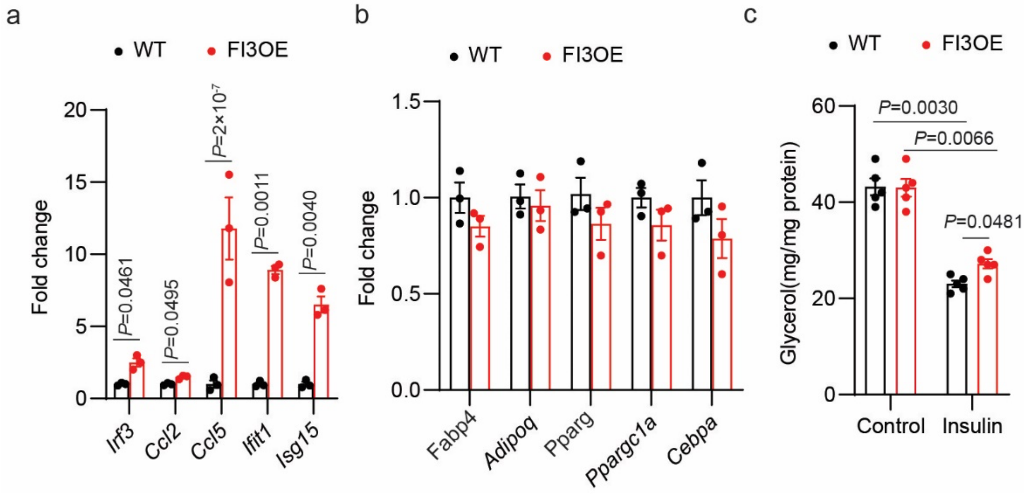

Supplementary Fig. 2. Overexpression of IRF3 does not affect differentiation state in mature adipocytes. a, mRNA analysis of *Irf3*, *Ccl5*, *Ifit1*, and *Isg15* in WT and FI3OE SVF-derived adipocytes (n=5). **b**, mRNA analysis of *Fabp4*, *Adipoq*, *Pparg*, *Ppargc1a*, and *Cebpa* in WT and FI3OE SVF-derived adipocytes (n=5). **c**, Lipolysis in WT and FI3OE SVF-derived adipocytes treated with or without insulin (10nM, 4h) (n=5). Statistical significance was assessed by two-tailed Student's *t-test* (**a**) or *three-way* ANOVA (**c**). Data in all panels are expressed as mean ± SEM.

Supplemental Figure 3

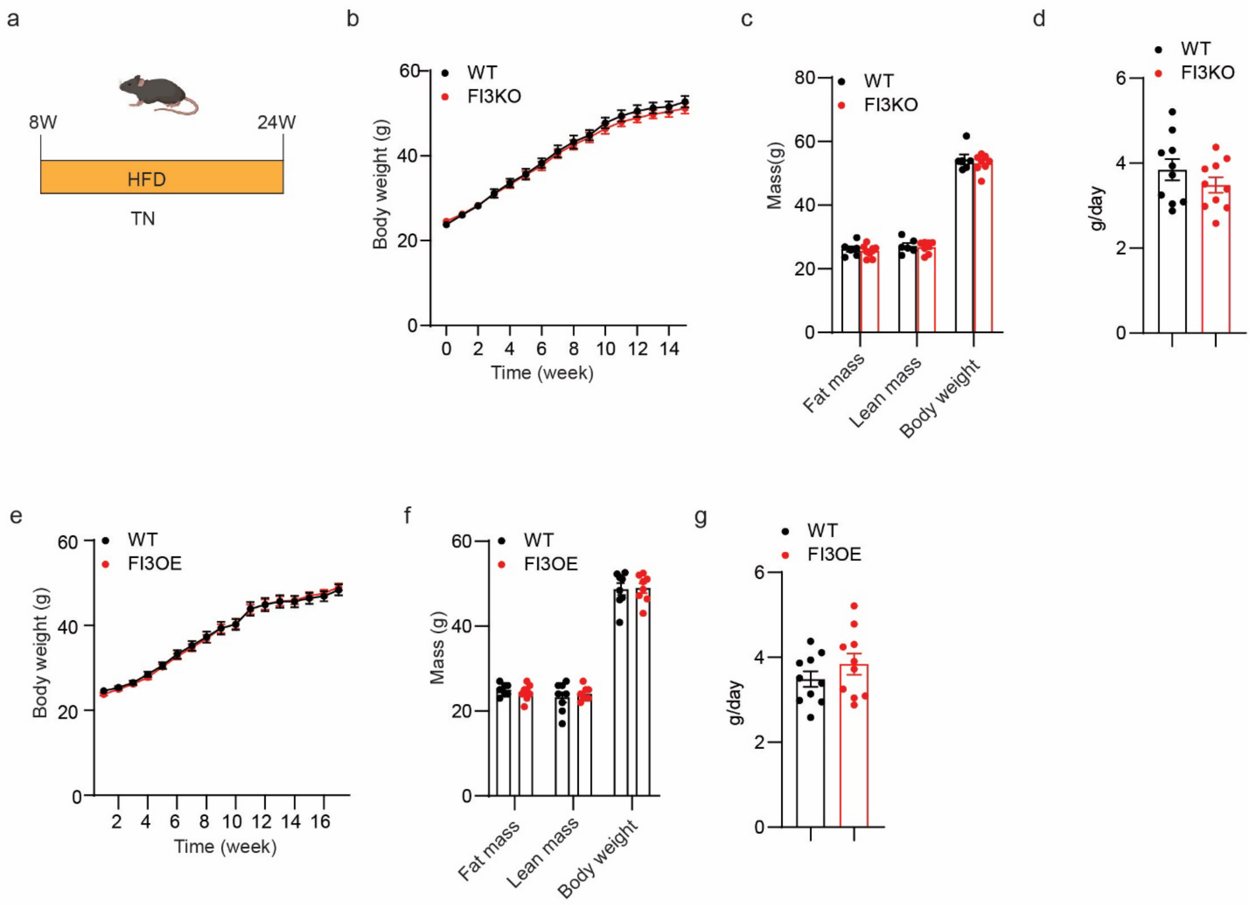

Supplementary Fig. 3. Metabolic phenotype of FI3KO and FI3OE mice on HFD in thermoneutrality. (**a**-**d**) Metabolic analysis of mice as described in Fig. 3**a**, body weight (**b**), body composition (**c**), and food intake (**d**). (**e**-**g**) Metabolic analysis of mice as described in Fig. 4**a**, body weight (**e**), body composition (**f**), and food intake (**g**). Mouse in Panel A created with Biorender.com.

Supplemental Figure 4

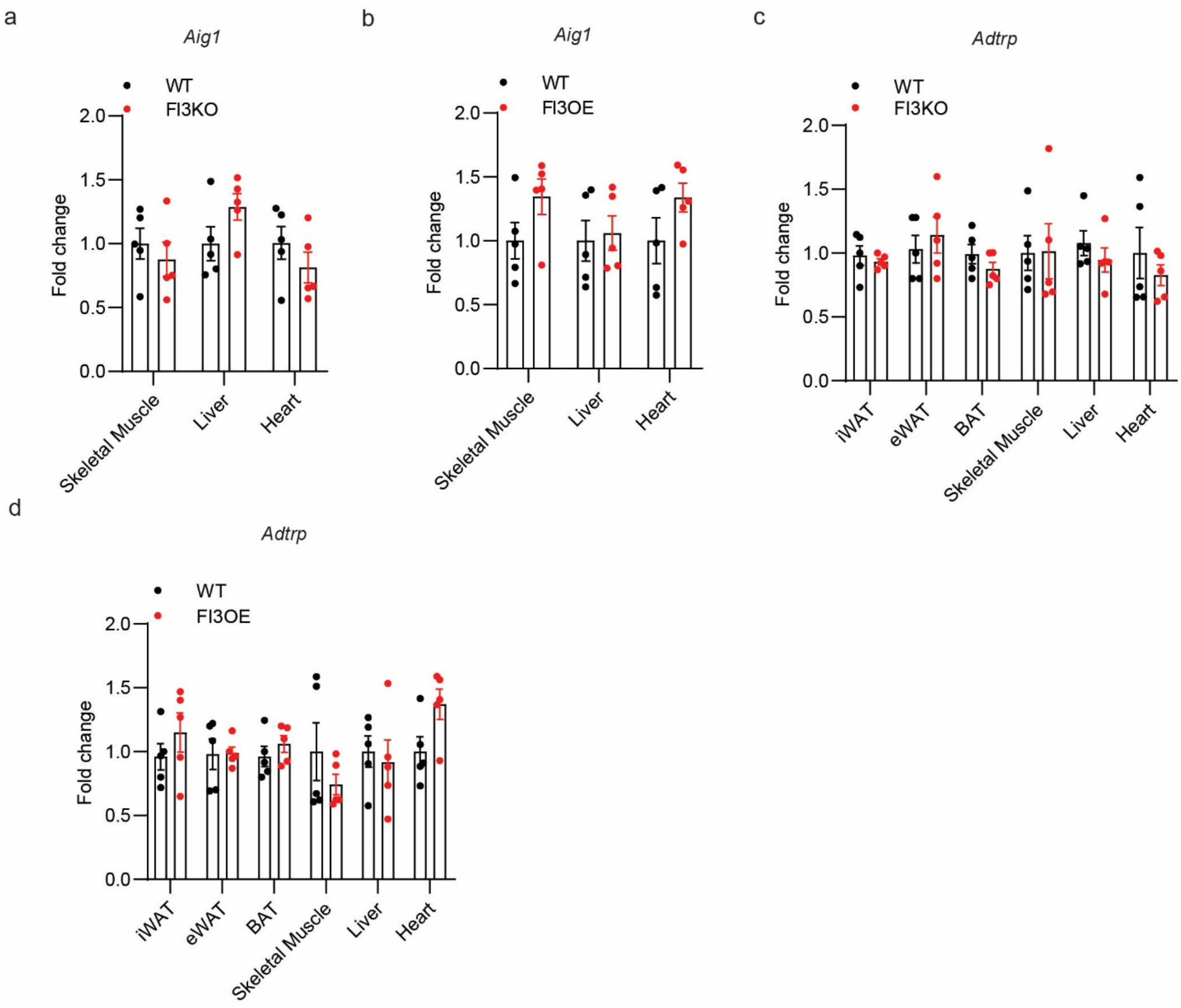

Supplementary Fig. 4. IRF3 regulates AIG1 transcription in adipocytes. a, mRNA analysis of *Aig1* in skeletal muscle, liver, and heart of WT and FI3KO mice (n=5). **b**, mRNA analysis of *Aig1* in skeletal muscle, liver, and heart of WT and FI3OE mice (n=5). **c**, mRNA analysis of *Adtrp* in skeletal muscle, liver, and heart of WT and FI3KO mice (n=5). **d**, mRNA analysis of *Adtrp* in skeletal muscle, liver, and heart of WT and FI3OE mice (n=5).

Supplemental Figure 5

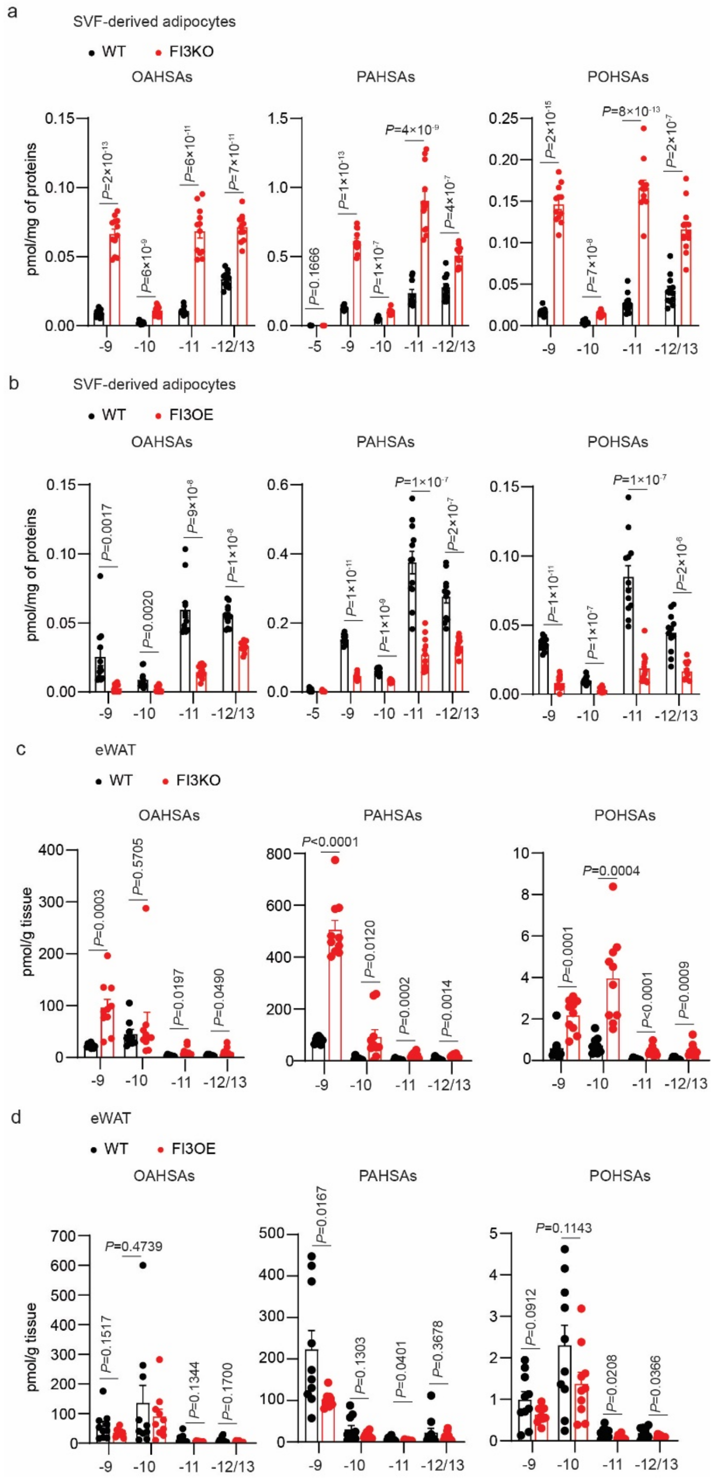

Supplementary Fig. 5. IRF3 decreases FAHFAs in adipocytes. a, Quantification of FAHFA isomers in WT and FI3KO SVF-derived adipocytes (n=12). **b**, Quantification of FAHFA isomers in WT and FI3OE SVF-derived adipocytes (n=12). **c**, Quantification of FAHFA isomers in eWAT of WT and FI3KO mice (n=10). **d**, Quantification of FAHFA isomers in eWAT of WT and FI3OE mice (n=10). Statistical significance was assessed by two-tailed Student's *t-test*. Data in all panels are expressed as mean ± SEM.

Supplemental Figure 6

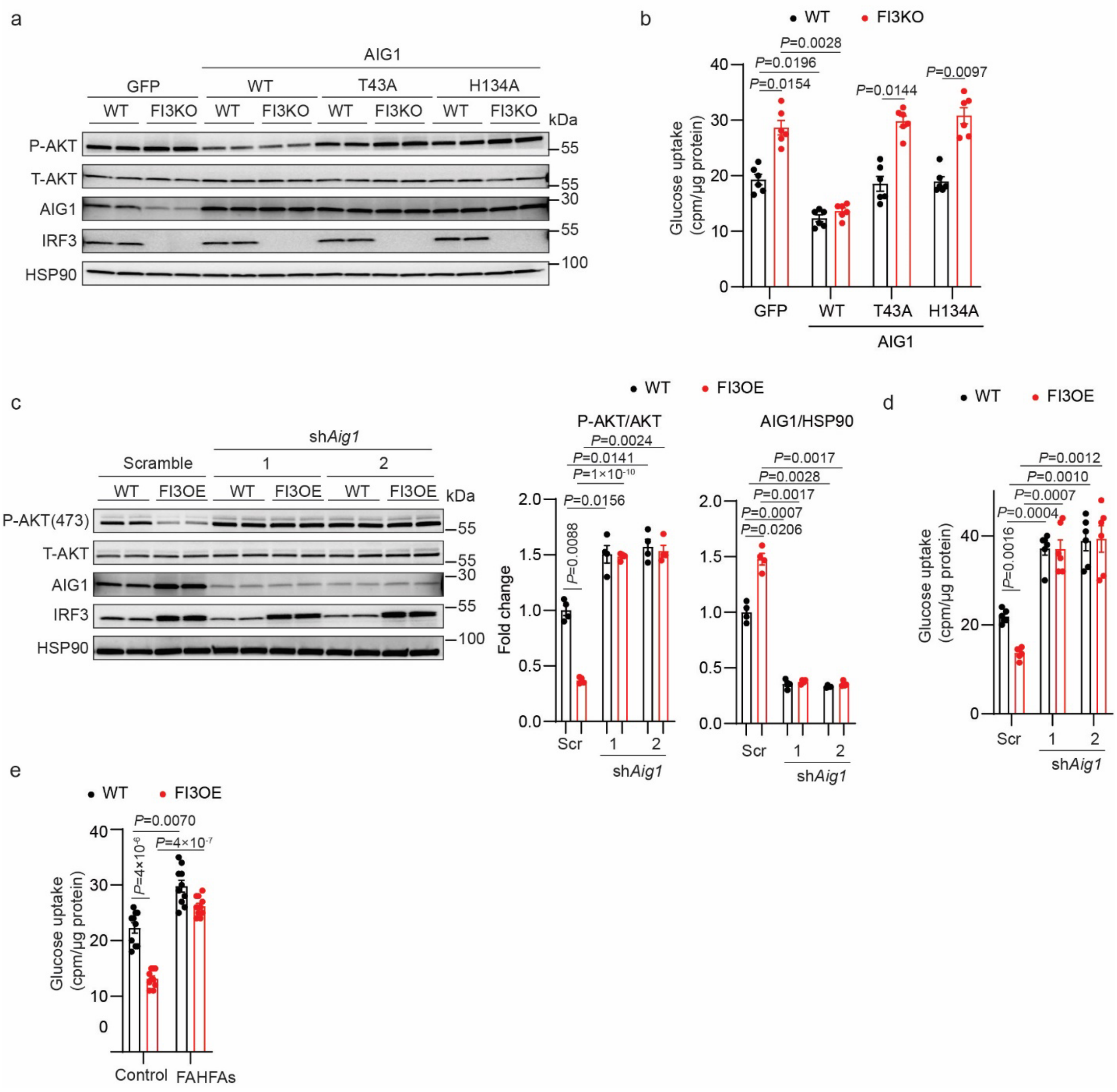

Supplementary Fig. 6. IRF3 promotes insulin resistance through AIG1 in adipocytes. a, Western blot of pAKT (S473) and IRF3, all lanes in the presence of insulin, **b**, Insulin-stimulated glucose uptake (n=6) in WT and FI3KO SVF-derived adipocytes infected with GFP, WT AIG1, T43A AIG1, or H134A AIG1 lentivirus. **c**, Western blot of pAKT (S473) and IRF3, **d**, Insulin-stimulated glucose uptake (n=6) in WT and FI3OE SVF-derived adipocytes infected lentivirus expressing shRNA against *Aig1* or shScr control hairpin. **e**, Insulin-stimulated glucose uptake in WT and FI3OE SVF-derived adipocytes treated with control or FAHFAs [9-PAHSA (20μM), 9-POHSA (20μM), and 9-OAHSA (20μM); total FAHFA levels 60μM] for 24h. Statistical significance was assessed by *two-way* ANOVA (b-e). Data in all panels are expressed as mean ± SEM.

Supplemental Figure 7

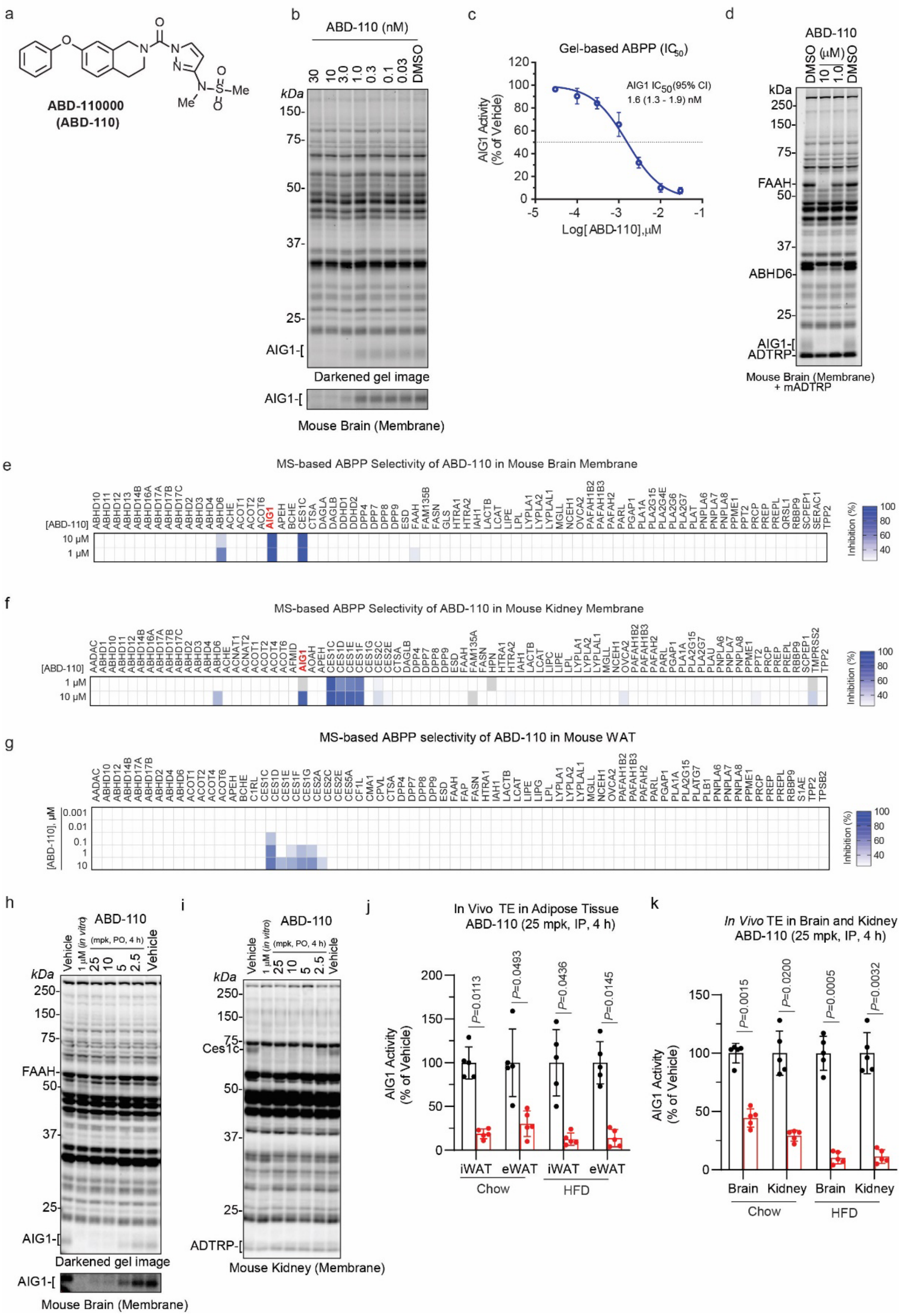

Supplementary Fig. 7. Characterization of the AIG1 inhibitor ABD-110000. a, Chemical structure of ABD-110000 (ABD-110). **b**, Competitive gel-based ABPP profile of ABD-110 (0.03–30 nM, 30 min) in mouse brain membrane proteome using FP-Rh. **c**, ABD-110 potency (IC50) for AIG1 determined by competitive

gel-based ABPP. Error bars represent SD from three replicates. **d**, Selectivity of ABD-110 (1 & 10 μM, 30 min) against FP-Rh-reactive enzymes in mouse brain membrane proteomes spiked with mADTRP-transfected HEK293T cell proteomes. **e**, **f**, In-depth selectivity profile of ABD-110 (1.0 and 10 μM) across serine/threonine hydrolase enzymes using MS-based ABPP profiles in mouse brain membrane (**e**) and kidney membrane (**f**) proteomes. Data presented represent mean inhibition from three replicates. Gray indicates targets that were not detected in any replicate. **g**, In-depth selectivity profile of ABD-110 (0.001-10 μM, 1 h) across serine hydrolase enzymes using MS-based ABPP profiles in Chow-fed mouse eWAT proteomes. Data presented represent mean inhibition from three replicates. **h**, **i**, Gel-based ABPP profiles in brain (**h**) and kidney (**i**) membrane proteomes derived from C57Bl/6J mice treated with either vehicle or ABD-110 (2.5 - 25 mpk, PO) for 4 h. Selective AIG1 inhibition is observed in brain whereas kidney profiles, where AIG1 is not detectable, reveal high selectivity across additional peripheral serine hydrolases and ADTRP. **j**, **k**, *In vivo* AIG1 target engagement following ABD-110 administration. Chow or HFD-fed mice were dosed with vehicle or ABD-110 (25 mpk, IP) once daily for 2 weeks. Brain, kidney, eWAT, and iWAT tissues were harvested four hours after the final dose for MS-based ABPP analysis. AIG1 activity was determined by measuring the degree of activity-dependent AIG1 enrichment relative to the vehicle using parallel reaction monitoring (PRM) to detect and quantify diagnostic AIG1 peptides. Statistical significance was assessed by two-tailed Student's *t-test*.

Supplemental Figure 8

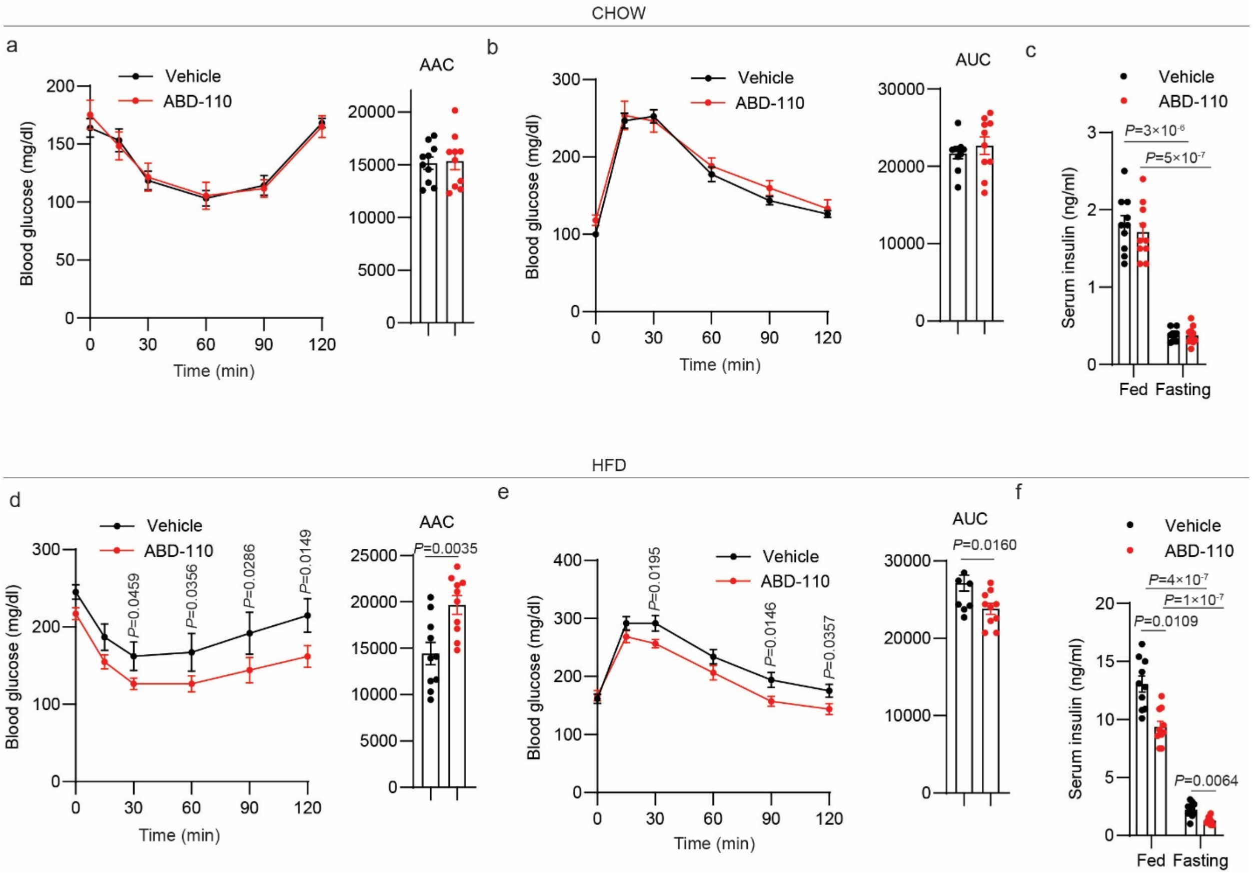

Supplementary Fig. 8. Metabolic effects of ABD-110 on WT Chow and HFD mice. Metabolic analysis of male WT mice (n=10) dosed with vehicle or ABD-110 (25 mg/kg IP) once daily for 2 weeks, including insulin tolerance test (**a**), glucose tolerance test (**b**), *ad lib* fed serum insulin levels (**c**). Metabolic analysis of male WT mice (n=10) after 16 weeks of HFD feeding dosed with vehicle or ABD-110 (25 mg/kg IP) once daily for 2 weeks, including insulin tolerance test (**d**), glucose tolerance test (**e**), *ad lib* fed serum insulin levels (**f**). Statistical significance was assessed by *two-way* ANOVA (**c** and **f**) or two-tailed Student's *t-test* (**d** and **e**). Data in all panels are expressed as mean ± SEM.

Supplemental Figure 9

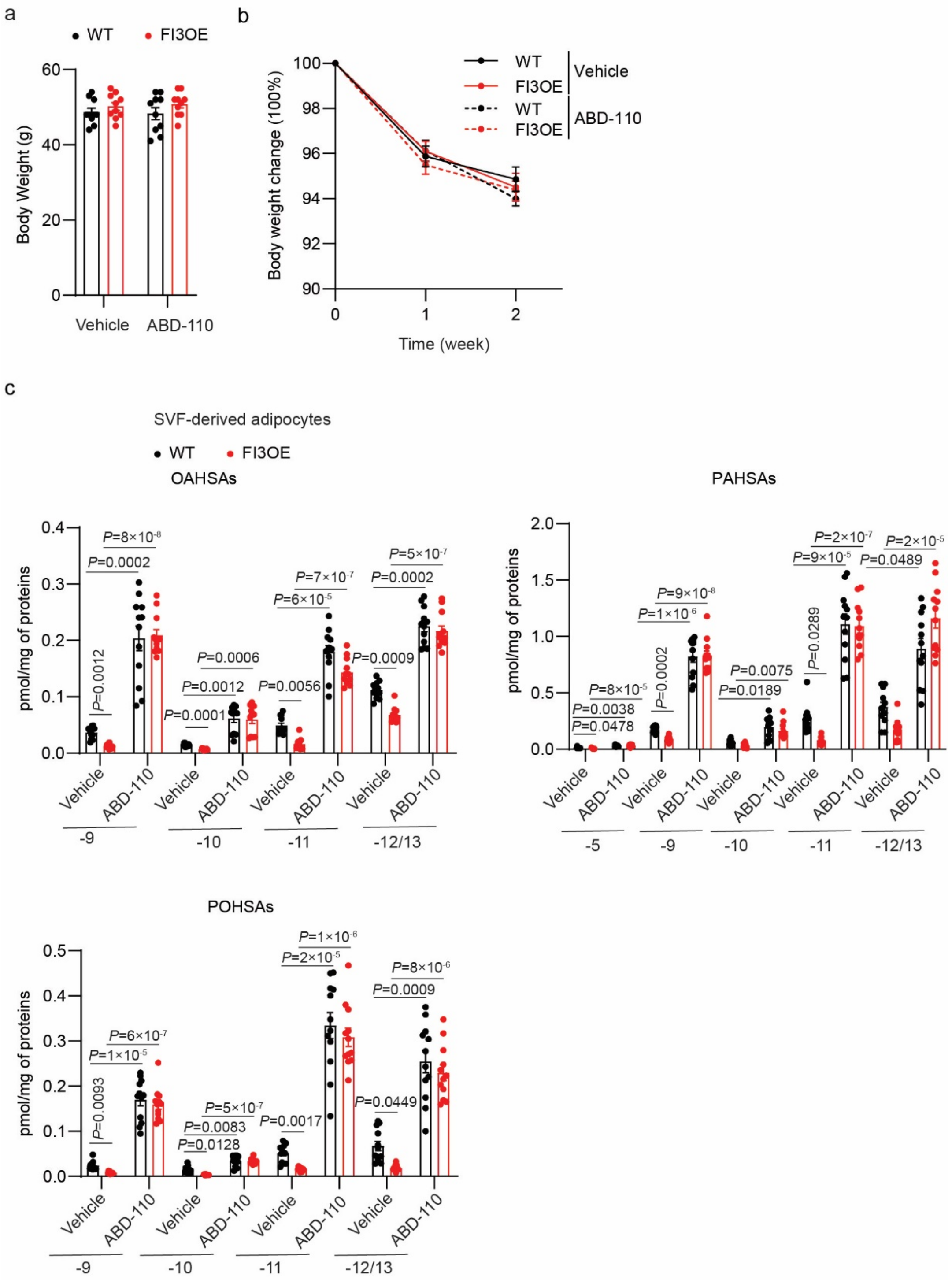

Supplementary Fig. 9. Inhibition of AIG1 by ABD-110 ameliorates HFD-induced insulin resistance in FI3OE mice. a, Body weight change of WT and FI3OE mice receiving a daily injection of vehicle or ABD-110 for two weeks (n=7-8). **b**, Body weight of WT and FI3OE mice after receiving a daily injection of vehicle or ABD-110 for two weeks (n=7-8). **c**, Quantification of FAHFA isomers in WT and FI3OE SVF-derived adipocytes treated with or without ABD-110 (1μM, 4h) (n=12). Data in all panels are expressed as mean ± SEM.

Supplemental Figure 10

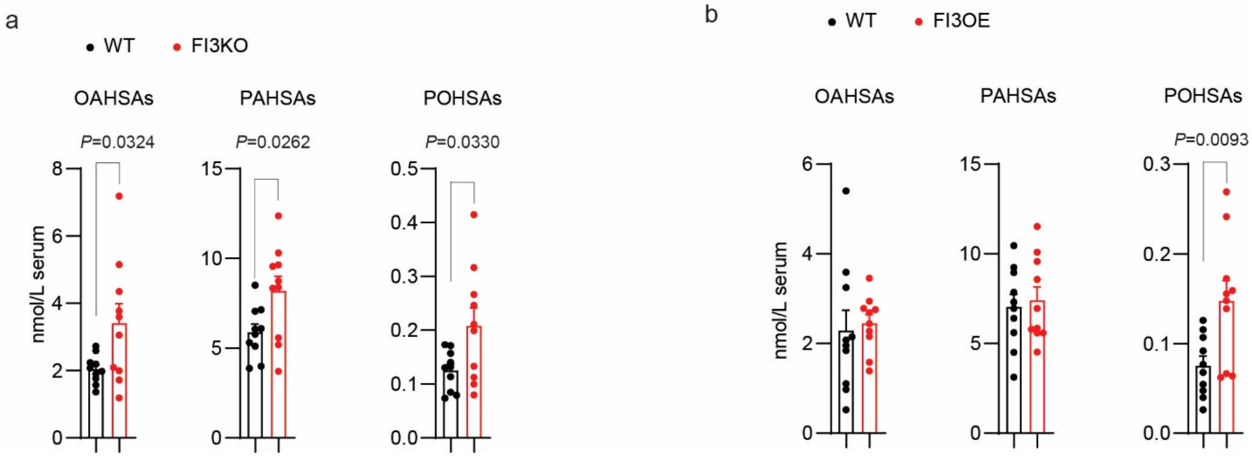

Supplementary Fig. 10. IRF3 regulates serum levels of some FAHFAs. a, Serum levels of OAHSAs, PAHSAs, and POHSAs in high fat-fed (16 weeks) FI3KO mice at thermoneutrality. **b**, Serum levels of OAHSAs, PAHSAs, and POHSAs in high fat-fed (16 weeks) FI3OE mice at thermoneutrality.

Supplementary Table 1

Primers used in this study

Genes (mouse)	Forward (5'-3')	Reverse (5'-3')
36B4	CACTGGTCTAGGACCCGAGAA	AGGGGGAGATGTTCAGCATGT
Aig1	ACCCAATCTTCTCTAAGCTGTGC	TCCCACCTTCCAGCATGAATG
Irf3	CGTACATCTGGGTGCCTCTC	TTTTCTTGGGGTGCAGGGTT
Ccl5	GCTGCTTTGCCTACCTCTCC	TCGAGTGACAAACACGACTGC
Ifit1	CAAGGCAGGTTTCTGAGGAG	TGAAGCAGATTCTCCATGACC
Isg15	CATCTATGAGGTCTTTCTGACGC	TTAGGCCATACTCCCCCAGC
Ap2	ACACCGAGATTTCCTTCAAACTG	CCATCTAGGGTTATGATGCTCTTCA
Adipoq	TGTTCCTCTTAATCCTGCCCA	CCAACCTGCACAAGTTCCCTT
Ppargc1a	CCCTGCCATTGTTAAGACC	TGCTGCTGTTCCTGTTTTC
Cebpα	TGGCCTGGAGACGCAATGA	CGCAGAGATTGTGCGTCTTT
Fabp4	AAGGTGAAGAGCATCATAACCCT	TCACGCCTTTCATAACACATTCC
Adtrp	TCACATCCCACAGATTGGAAGG	AATGGCCTGCAAGAGCAGATT

Supplementary Table 2

Peptides targeted by parallel reaction monitoring to measure AIG1 target engagement in vivo.

PROTEIN	PEPTIDE SEQUENCE	M/Z	Z
Aig1	AIEM[+16]PSHQTYGGSWK	569.9314	3
Aig1	EM[+16]IYPR	412.7022	2
Fasn	VGDPQELNGITR	649.8386	2
Fasn	LFDHPEVPTPPESASVSR	655.6619	3
Fasn	GVDLVLNSLAEEK	693.8774	2
Pcca	TVAIHSDVDASSVHVK	555.6249	3
Pcca	FLSDVYPDGFK	644.3164	2
Pc	SLPDLGLR	435.7558	2
Pc	DFTATFGPLDSLNTR	827.9072	2

Supplementary Note 1: Lipidomics Checklist

Separation Workflow

Created by https://lipidomicstandards.org, version v2.3.2

Overall study design

Title of the study	Inflammation causes insulin resistance via interferon regulatory factor 3 (IRF3)-mediated reduction in FAHFA levels		
Document creation date	01/11/2024	Corresponding Email	erosen@bidmc.harvard.edu
Principle investigator	Evan Rosen and Barbara B. Kahn	Is the workflow targeted or untargeted?	Targeted
Institution	Beth Israel Deaconess Medical Center, Harvard Medical School	Clinical	No

Lipid extraction

Extraction method	Solid-phase extraction only for eWAT and serum	Were internal standards added prior extraction?	Yes
pH adjustment	None		

Analytical platform

Which solvents were used	93:7 methanol/water with 5mM ammonium acetate and 0.01% ammonium hydroxide	Ion source	ESI
Number of separation dimensions	One dimension	MS Level	MS2
Separation type 1	LC	Mass window for precursor ion isolation (in Da total isolation window)	1
Separation mode 1 (liquid)	RP	Mass resolution for detected ion at MS2	Low resolution
Detector	Mass spectrometer	Resolution at MS2	Low
MS type	QQQ	Was/Were additional dimension/techniques used	No
MS vendor	Agilent		

Quality control

Blanks	Yes	Quality control	Yes
Type of Blanks	Extraction blank, Injection blank	Type of QC sample	Reference material

Method qualification and validation

Method validation	Yes	Precison	Yes
Lipid recovery	No	Accuracy	Yes
Dynamic quantification range	No	Guidelines followed	This method was published in Nature Protocols (Zhang et al., 2016)
Limit of quantitation (LOQ)/Limit of detection (LOD)	Yes		

Reporting

Are reported raw data uploaded into repository?	No	Raw data upload	Yes
Are metadata available?	Available on request	Additional comments	-

Sample Descriptions

WT and IRF3 KO Stromal Vascular Fraction (SVF) derived adipocytes / Mouse / Cells

Provided information	-	Additives	None
Temperature handling original sample	4-8 °C	Were samples stored under inert gas?	No
Instant sample preparation	No	Additional preservation methods	No
Storage temperature	-80 °C	Biobank samples	No

WT and IRF3 overexpressing SVF derived adipocytes / Mouse / Cells

Provided information	-	Additives	None
Temperature handling original sample	4-8 °C	Were samples stored under inert gas?	No
Instant sample preparation	No	Additional preservation methods	No
Storage temperature	-80 °C	Biobank samples	No

epidydimal White Adipose Tissue (eWAT) samples from WT and IRF3 KO mice / Mouse / Tissues (e.g., liver, heart, brain)

Perfusion	No	Were samples stored under inert gas?	No
Provided information	-	Additional preservation methods	No
Temperature handling original sample	4-8 °C	Biobank samples	No
Instant sample preparation	No	Sample homogenization	Yes
Storage temperature	-80 °C	Sample homogenization solvent	PBS:Methanol:Chloroform (plus Internal Standards) 1:1:2
Additives	None		

eWAT samples from WT and IRF3 overexpressing mice / Mouse / Tissues (e.g., liver, heart, brain)

Perfusion	No	Were samples stored under inert gas?	No
Provided information	-	Additional preservation methods	No
Temperature handling original sample	4-8 °C	Biobank samples	No
Instant sample preparation	No	Sample homogenization	Yes
Storage temperature	-80 °C	Sample homogenization solvent	PBS:Methanol:Chloroform (plus Internal Standards) 1:1:2
Additives	None		

serum from WT and IRF3 KO mice / Mouse / Serum

Provided information	-	Additives	None
Temperature handling original sample	4-8 °C	Were samples stored under inert gas?	No
Instant sample preparation	No	Additional preservation methods	No
Storage temperature	-80 °C	Biobank samples	No

serum from WT and IRF3 overexpressing mice / Mouse / Serum

Provided information	-	Additives	None
Temperature handling original sample	4-8 °C	Were samples stored under inert gas?	No
Instant sample preparation	No	Additional preservation methods	No
Storage temperature	-80 °C	Biobank samples	No

SVF derived adipocytes from WT and IRF3 overexpressing mice +/-AIG1 inhibitor / Mouse / Cells

Provided information	-	Additives	None
Temperature handling original sample	4-8 °C	Were samples stored under inert gas?	No
Instant sample preparation	No	Additional preservation methods	No
Storage temperature	-80 °C	Biobank samples	No

Lipid Class Descriptions

1) FAHFA[M-H]- / Lipid identification

Lipid class	FAHFA	Check isomer overlap	No
Derivatization	-	RT verified by standard	Yes
MS Level for identification	MS2	Separation of isobaric/isomeric interferece confirmed	Yes
Identification level	Species level	Model for separation prediction	Yes
Polarity mode	Negative	Additional dimension/techniques	-
Type of negative (precursor)ion	[M-H]-	Lipid Identification Software	Qualitative Analysis of MassHunter Acquisition Data (Agilent)
Fragments for identification		Data manipulation	Smoothing

Fragment name
PAHSA m/z 537.5 → m/z 255.2
PAHSA m/z 537.5 → m/z 281.2
OAHSA m/z 563.5 → m/z 281.2
OAHSA m/z 563.5 → m/z 299.3
POHSA m/z 535.5→ m/z 299.3
POHSA m/z 535.5→ m/z 253.2
POHSA m/z 535.5→ m/z 271.3
POHSA m/z 535.5→ m/z 281.2

Isotope correction at MS2	No	Nomenclature for intact lipid molecule	No
MS2 verified by standard	Yes	Nomenclature for fragment ions	No
Background check at MS2	No	Further identification remarks	-
Did you presume assumptions for identification?	No		

1) FAHFA[M-H]- / Lipid quantification

Quantitative	Yes	Limit of quantification	S/N ratio
MS Level for quantification	MS2	Normalization to reference	No
Internal lipid standard(s) MS2		Lipid Quantification Software	Qualitative Analysis of MassHunter Acquisition Data (Agilent)

Internal standard	Fragment(s)	Endogenous subclass
13C16-9-PAHSA	m/z 553.5 → m/z 271.3	PAHSA and POHSA
13C16-5-PAHSA	m/z 553.5 → m/z 271.3	PAHSA
13C18-12-OAHSA	m/z 581.6 → m/z 299.3	OAHSA

Type of quantification	Internal standard amount	Batch correction	No
Response correction	No	Further quantification remarks	-
Type I isotope correction	No		
