# Supplementary material for: Inflammation causes insulin resistance in mice via interferon regulatory factor 3 (IRF3)-mediated reduction in FAHFA levels
Source: Nat Commun. 2024 May 30;15:4605. doi: 10.1038/s41467-024-48220-5 (PMC11139994; doi:10.1038/s41467-024-48220-5)
Supplement: Supplementary file 3 — Description of Additional Supplementary Files [file 41467_2024_48220_MOESM3_ESM.pdf]

<u>Description of Additional Supplementary Files</u>

Supplementary Data 1: Gene expression in adipocytes expressing IRF3-2D vs. GFP (associated with main Figure 5). Fold change calculated as mean in IRF3-2D over mean in GFP.
